# Case Report: a newly described form of fixed subaortic stenosis in a feline patient

**DOI:** 10.3389/fvets.2025.1672336

**Published:** 2025-12-02

**Authors:** Maria Cerbu, Irina Constantin, Iuliu-Călin Scurtu, Constantin Cerbu, Alexandru-Flaviu Tăbăran

**Affiliations:** 1 Department of Comparative Anatomy, Faculty of Veterinary Medicine, University of Agricultural Sciences and Veterinary Medicine, Cluj-Napoca, Romania; 2 Department of Veterinary Pathology, Faculty of Veterinary Medicine, University of Agricultural Sciences and Veterinary Medicine, Cluj-Napoca, Romania; 3 Department of Internal Medicine, Faculty of Veterinary Medicine, University of Agricultural Sciences and Veterinary Medicine, Cluj-Napoca, Romania; 4 Department of Infectious Diseases, Faculty of Veterinary Medicine, University of Agricultural Sciences and Veterinary Medicine, Cluj-Napoca, Romania

**Keywords:** fixed subaortic stenosis, cushion-like pseudovalve, rare, feline, case report

## Abstract

Fixed subaortic stenosis as a congenital heart defect has been reported in a relatively small number of cases. The classic description of feline subaortic stenosis reflects the presence of a fibrous ring just underneath the aortic valve. The present case has a unique morphological aspect, analogous with a rare human stenosis. A 15-month-old, ESH female presented with an anamnesis consisting of respiratory distress, apathy, and anorexia. The clinical examination displayed a patient with lethargy and severe dyspnea, whilst lung auscultation identified generalized bilateral pulmonary crackles. A systolic IV/VI parasternal murmur was identified on the left thorax, while the murmur intensity on the right side was III/VI. Electrocardiogram showed a sinus rhythm interrupted by ventricular ectopic beats. Two-dimensional (2D) echocardiography revealed a concentric hypertrophy of the left ventricle, with severe left atrium enlargement and mild pleural and pericardial effusion. A subaortic, hyperechoic structure was seen from the right parasternal five-chamber view, accompanied by post-stenotic aortic dilatation. The aortic peak velocity was 5.2 m/s, a feature that characterizes the severe form of aortic stenosis. The cat died shortly after presentation and necropsy was performed. Subaortic stenosis was confirmed on gross examination. The structure that induced stenosis of the left ventricle outflow tract (LVOT) had the appearance of a rudimental valve with moderator bands attached. Microscopically, it contains fibrous and smooth muscular tissue and cardiac conduction fibers. This type of subaortic stenosis resembles the cushion-like pseudovalve type described only in human cardiology. This is the first case describing clinical signs, ECG, echocardiography, and histopathological findings in a cat with cushion-like pseudovalve subaortic stenosis. Fixed subaortic stenosis is a rare congenital disease in cats, which should be taken into consideration in young cats with dyspnea and left ventricular hypertrophy.

## Introduction

Aortic stenosis (AS) is a rare congenital heart defect in cats. It is represented as a narrowing of the left ventricular outflow tract (LVOT) and/or the aorta, located below the aortic valve (subvalvular stenosis), above it (supravalvular stenosis), or at the valve level (valvular stenosis). This narrowing produces a turbulent blood flow that can be heard as a systolic murmur on the left heart base, as well as an increased blood flow velocity that can be detected and measured by Doppler echocardiography (1).

In dogs, aortic stenosis is one of the most frequently diagnosed congenital heart pathology (1–5). Based on post-mortem changes, AS is classified as a mild form, when small, whitish, slightly raised nodules are present on the endocardium of the ventricular septum, just below the aortic valve; a moderate form characterized by the presence of a ridge of fibrous tissue which extends from the base of the anterior leaflet of mitral valves across the interventricular septum to underneath the left coronary cusp; a severe stenosis characterized by the presence of a fibrous band or ridge, which completely encircles the left ventricular outflow tract below the aortic valve and forms a concentrically narrowing tunnel (6).

Although no consensus, there is a generally accepted severity scale where a patient is considered to be SAS-affected with an aortic velocity greater than 2.5 m/s. Based on the aortic velocity cutoffs and pressure gradients, respectively, aortic stenosis has been graded as a. equivocal (2–2.5 m/s; 16–24 mm Hg), b. mild (2.5–3.5 m/s; 25–49 mm Hg), c. moderate (3.5–4.5 m/s; 50–79 mm Hg), and d. severe (>4.5 m/s; ≥80 mm Hg) (3, 7).

Based on functional characteristics of the obstruction, subvalvular aortic stenosis is further categorized as fixed (static or resting) or dynamic (labile) (7, 8). A dynamic form of subaortic stenosis can occur in the following events: in idiopathic hypertrophic left ventricle with the ventricular septum protrusion into the LVOT; systolic anterior motion of the mitral valve (SAM), frequent in cats, which can occur concurrently or in the absence of LVH; in cases where aortoseptal angle is smaller than 180° from various reasons (9). The typically described features of feline fixed subaortic stenosis reflect the presence of a fibrous ring just underneath the aortic valve, encircling the LV outflow tract. This tissue may take the form of a discrete thin band, or as a wide plaque-like proliferation (10). This can cause left ventricular outflow tract obstruction, with concomitant increased afterload and compensatory left ventricular concentric hypertrophy and left atrial enlargement (11).

### Patient information and clinical findings

A 15-month-old female European Short Hair (ESH) cat, weighing 3 kg, was brought to the Veterinary Emergency Hospital from the Faculty of Veterinary Medicine Cluj-Napoca. The cat has been experiencing respiratory issues, worsening over the past 3 days, apathy, and anorexia for the last 48 h. Upon examination, the cat exhibited a good body condition score (5/9), mild dehydration (5%), and dyspnea. A severe respiratory distress with abdominal effort developed during the clinical examination, and pulmonary crackles were detected upon auscultation. Blood work-up was carried-out with the evidence of a mild respiratory acidosis (pH 7.2, pCO_2_ 45.8 mmHg, HCO_3_ 22.6 mmol/L, and lactate 3.2 mmol/L). The cat was tested for the most common viral feline infectious disease and was positive for Feline Coronavirus Antigen Test (Vet Expert rapid test). Thoracic radiography confirmed generalized lung edema and the cat was referred to the Cardiology Department for further evaluation.

**Figure 1:**
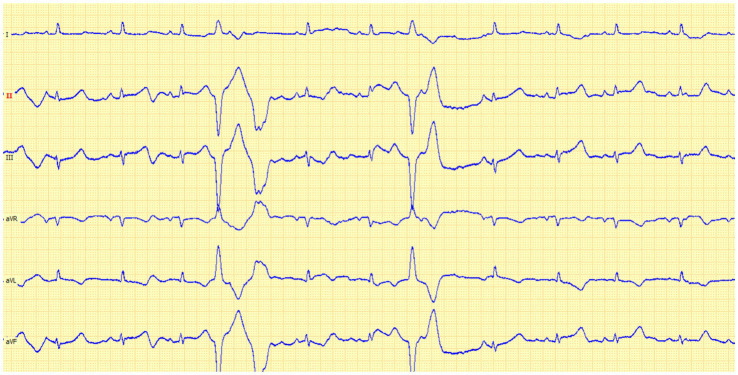
Six lead ECG in a 15-month-old cat identified a sinus rhythm interrupted by two ventricular premature complexes with right-bundle branch block (RBB) morphology, suggesting left ventricle origin.

On heart auscultation, left parasternal IV/VI systolic murmur was identified, while murmur intensity on the right side was III/VI. Diffuse pulmonary crackles were identified bilaterally. The femoral pulse was considered normal. ECG revealed a sinus rhythm interrupted by isolated ventricular beats with a heart rate of 120 p.m. (Figure 1).

### Diagnostic assessment

**Figure 2:**
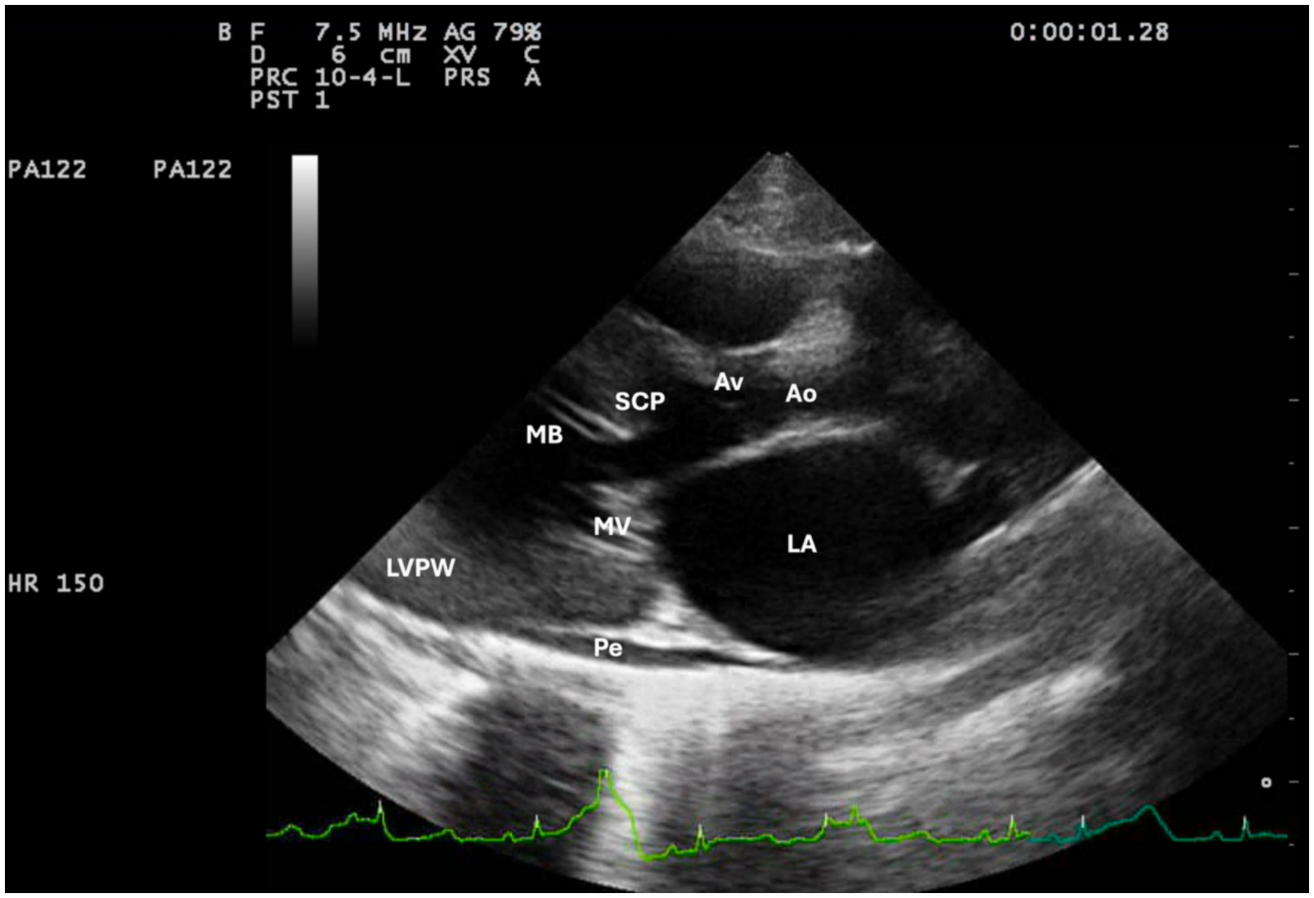
Echocardiographic for right parasternal long-axis five-chamber view. LA, left atrium; LVPW, left ventricle posterior wall; Ao, aorta; Av, aortic valves; SCP, subaortic cushion-like pseudovalve; MV, mitral valve; MB, moderator bands; Pe, pericardial effusion.

The standard transthoracic 2D echocardiography (12) identified in the right-parasternal, long-axis four-chamber view, a concentric hypertrophy of the left ventricle. The interventricular septum diameter in end-diastole was 8 mm. In the right parasternal long-axis four-chamber view at end-systole, a moderate left atrium dilatation was identified, with a left atrium transversal diameter (LAD) of 20 mm, and mild pleural and pericardial effusion were observed. In the right parasternal, long-axis five-chamber view, color Doppler identified a turbulence within left ventricle outflow tract and a mitral regurgitation resembling with the classic aspect of systolic anterior motion of the septal mitral leaflet (SAM). In the same view, beneath the aortic valve, a subaortic hyperechogenic structure was noticed (Figure 2). Post-stenotic aortic dilatation was also present. On the five-chamber view from left-side, the aortic peak velocity was 5.2 m/s, which corresponds to a severe aortic stenosis (3).

Despite specific therapy, consisting of oxygen (via box), buthorphanol (0.2 mg/kg, IV), and furosemide (2 mg/kg IV), the cat died in a short time after presentation.

**Figure 3:**
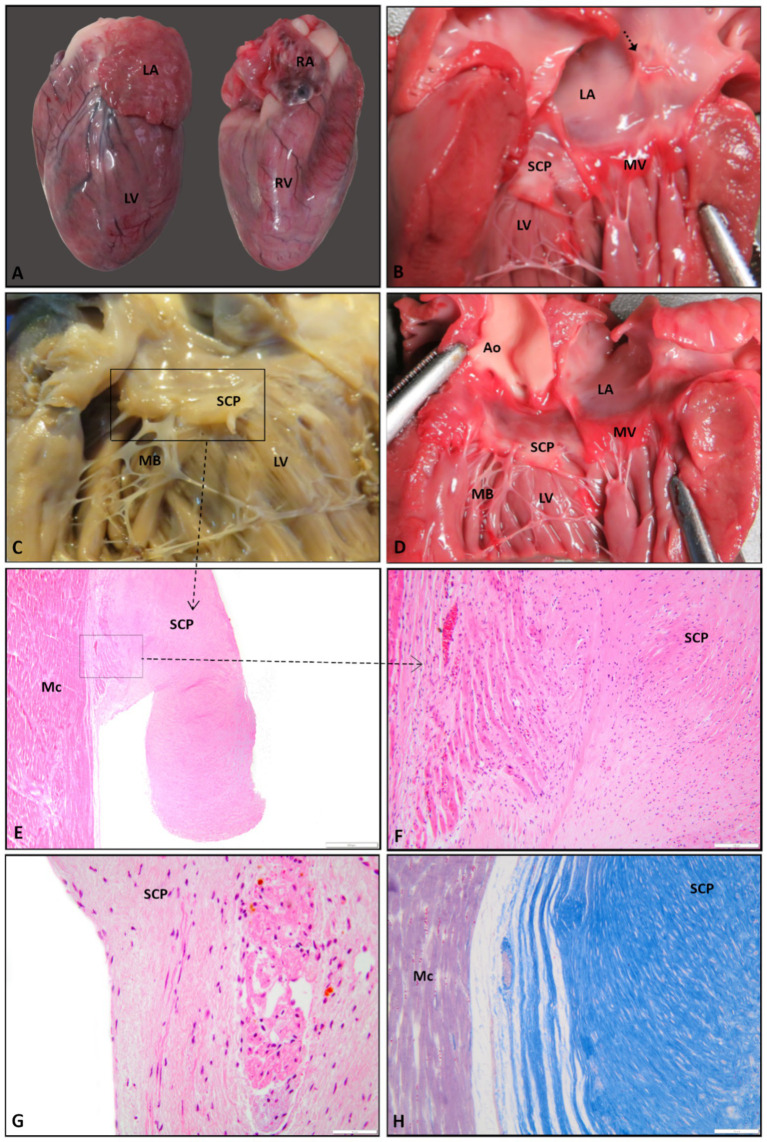
Gross and histological features of the cushion-like pseudovalve: **(A)** the heart is markedly enlarged, with elongation of the long axis and severe, diffuse distention of the left atrium–left ventricle and right ventricle faces; **(B)** the subaortic cushion-like pseudovalve and the fibrosis of the left atrial endocardium (black arrow); **(C)** the moderator bands connect the subaortic cushion-like pseudovalve with both free and septal ventricular walls (fixed specimen); **(D)** frontal view of the subaortic cushion-like pseudovalve located under the mitral valve, with few rudimentary chords attaching the pseudovalve to the left ventricle; **(E, F)** histologically the subaortic pseudovalve consists of fibrous connective tissue composed of parallel bundles of thick collagen separated by few fibrocytes and pale-basophilic, myxoid extracellular matrix. Fibrous connective tissue surrounding the cardiomyocytes and foci of metaplastic cartilage (H&E stain, Image E bar = 500 μm, Image F bar = 100 μm); **(G)** glycogen-rich cardiomyocytes, H&E stain, bar = 50 μm; **(H)** predominance of the fibrous connective tissue within the structure of the pseudovalve, Masson’s trichrome stain, bar = 100 μm. LA, left atrium; LV, left ventricle; RA, right atrium; RV, right ventricle; Ao, aorta; SCP, subaortic cushion-like pseudovalve; MV, mitral valve; MB, moderator bands; Mc, myocardium.

During necropsy, cardiogenic severe pulmonary congestion and edema (diffuse, bilateral) was present. The gross examination of the heart showed marked concentric hypertrophy of the left ventricle, with a total cardiac mass of 28 grams, which is 0.93% of the total body weight (3 kg). This hypertrophy was accompanied by severe dilation of the left auricle. The presence of marked stenosis in the left ventricular outflow tract (LVOT), characterized by the appearance of a rudimental valve resembling the description of the cushion-like pseudovalve (13) with moderator bands attached (Figures 3B–D), was observed. Moderate post-stenotic dilatation was noted at the level of the aorta. Additionally, within the left auricle adjacent to the mitral valve, a well-demarcated, focal-extensive area of fibrosis measuring 1 cm × 1 cm with partial endocardial disruption was present. The heart was fixed in 10% neutral buffered formalin (Figure 3C). The routine protocol of paraffin wax embedding was followed, and the samples were sectioned at 4 μm. The slides were stained with Hematoxylin and Eosin stain (H&E stain) (Figures 3E–G) and Masson’s trichrome stain (Figure 3H) (14). The myocardium showed concentric hypertrophy without myofiber disarray or diffuse interstitial fibrosis, features required for the diagnosis of primary HCM. The changes were consistent with pressure-overload hypertrophy secondary to a fixed subaortic obstruction. Microscopically, the cushion-like pseudovalve (Figure 3E) was composed of irregularly arranged bundles of cardiomyocytes interspersed with edematous fibrous connective tissue, foci of the metaplastic cartilage (Figure 3F), and glycogen-rich cardiomyocytes, resembling cells of the conduction system (Figure 3G) (1, 15). The predominance of the fibrous connective tissue within the composition of the pseudovalve was confirmed with Masson’s trichrome staining (Figure 3H).

Table 1 summarizes the progression of clinical signs, diagnostic evaluations, therapeutic interventions, and outcome.

**Table 1:** Timeline of clinical events, diagnostic findings, and interventions.

Time Point	Findings	Diagnostics	Interventions
Day −3	Respiratory distress	–	–
Day −2 to 0	Apathy, anorexia	–	–
Day 0 (admission)	Dyspnea, 5% dehydration, pulmonary crackles	Blood gas: pH 7.2, pCO₂ 45.8; FCoV +	O₂, butorphanol, furosemide
Day 0 (imaging)	Pulmonary edema	Thoracic radiographs	–
Day 0 (cardiology)	Murmur (IV/VI), arrhythmia	Echo: LV hypertrophy, LA dilation, LVOT obstruction, AV 5.2 m/s	–
After echo	Respiratory worsening	–	O₂, diuretic
Same day	Death	–	–
Post-mortem	Severe pulmonary edema, LV hypertrophy, LVOT stenosis	Histology: fibrous tissue, cartilage in pseudovalve	–

## Discussion

Subaortic stenosis is a rare congenital disease in cats (1, 8, 11, 16, 17), and although the numbers are sparse, subvalvular AS appears to be the most common form (10, 18–21). However, the scarcity of recent descriptions of this congenital heart disease makes it questionable nowadays if it is indeed a common disease (11). With only a few cases published recently (11, 16, 22, 23), SAS remains on top of congenital pathology in cats, being the second, after ventricular septal defect (2), and the sixth according to Tidholm (24). It is encountered in 7%–17% of all congenital cardiac defects in this species (25).

In humans, subaortic stenosis is divided in four categories: type I, a thin membranous diaphragm at the cranial portion of the left ventricular outflow tract; type II, a thick fibrotic ring, few centimeters below the aortic valve; type III, irregular fibromuscular additional tissue just below the aortic valve; and type IV, tunnel-like obstruction of the left ventricular outflow tract (26, 27). In only two cases, postmortem necropsy revealed a membranous or cushion-like pseudovalve that produced the obstructions of the left ventricular outflow tract (28). In our case, subaortic obstruction does not resemble the classical forms described in dogs and human (6), yet more with the cushion-like pseudovalve type.

Currently, diagnosis of stenosis is based on echocardiographic evaluation of the left ventricular outflow tract morphology and the measurement of LVOT/aorta peak velocity with continuous wave (CW) Doppler (1). In a study describing six cats with SAS (16), the diagnosis was confirmed by different methods: angiography, echocardiography, or necropsy. Only four out of six cats were further sent for a complete necropsy examination, and each of them had classic fibrous-appearing subaortic bands encircling and obstructing the left ventricular outflow tract.

The clinical signs reported in our case, especially dyspnea and pulmonary crackles, and systolic murmur with the point of maximum intensity on the left cardiac base, along with the echocardiography findings, such as LVH, LA dilation, turbulent jet on LVOT, elevated transvalvular pressure gradient at the level of the aortic artery, and post stenotic dilatation, are consistent findings found in all previously described feline subaortic stenosis (SAS) cases (2, 11, 16, 24, 29). The novelty of our case represents the ECG examination with abnormalities identified, consisting of ventricular premature contraction (VPC’s). Usually, on ECG, depression of the ST segment may appear due to a hypoxic, hypertrophied left ventricle, suggesting myocardial ischemia or secondary repolarization changes (1). However, in most cases, the ECG reveals a normal sinus rhythm (11, 16). A particular change identified in this case is the macroscopic appearance of the subaortic stenosis structure, a rudimental valve with a moderator bands attached, or a cushion-like pseudovalve, as described in human cardiology (13). This type of structure was described in two cases, and in only one, the histopathology was available, which confirmed the stenosis as being a fibrous pseudovalve. There are no other specifications, as in our case, there were also moderator bands attached or other tissues involved beside the fibrous one. Therefore, a clear definition or set of standard diagnostic criteria for this subtype of subaortic stenosis is lacking. However, it can be noted that this type of stenosis is distinguished by its macroscopic appearance, resembling a pseudovalve. The moderator bands are considered to be a congenital malformation (30), which appear as muscular tendons bundles, with the role of creating a connection between the ventricular septum and the free ventricular wall (31), and with a potential role to prevent excessive straining in ventricular diastole (32). Moderator bands are a common finding in mammals within the structure of the right ventricle, but they are considered rather rare in the left ventricle (31, 33).

SAS have hypertrophic cardiomyopathy as the primary differential diagnosis followed by secondary LVH, caused by systemic hypertension, acromegaly, myocardial infiltrative disease (e.g., lymphoma), hyperthyroidism, dystrophin-deficient hypertrophic feline muscular dystrophy, or administration of steroids (28, 34). The difficulty in confirming feline aortic stenosis lies in the high prevalence of HCM (28, 34, 35) with systolic anterior motion of the mitral valve. The mitral regurgitation noticed in some cases of subaortic stenosis may develop as the anterior mitral leaflet is drawn into the left ventricular outflow tract during the systole by the high velocity flow (16), which makes the diagnosis even more difficult. In cats with longstanding SAM or focal basilar septal hypertrophy, it may be impossible to be confident in a diagnosis of subaortic stenosis when high LVOT velocities are also visualized (17). AS shares many clinical and diagnostic features with idiopathic HCM, including left ventricular hypertrophy, mitral regurgitation, left atrial dilation, varying degrees of left ventricular outflow obstruction, high aortic blood flow velocity, and left-sided congestive heart failure (2, 11, 16, 24, 29).

In our case, the age of the patient and the appearance of a hyperechogenic structure just below the aortic valve, accompanied by a post-stenotic aortic dilatation, helped in certifying SAS as the diagnosis. Usually in ESH cats, HCM appears in middle-aged to older cats (36), and our patient was young, therefore the diagnosis was oriented toward a congenital pathology. In neither of the literature consulted was it specified that HCM can induce aortic dilatation and the hyperechogenic appearance below the aortic valve was the key element. Based on both the echocardiographic and histopathological findings, the possibility of concomitant hypertrophic cardiomyopathy in this case can be considered extremely unlikely.

As a clinical sign, dyspnea is the most common finding in cats with SAS or other types of AS (1, 11, 16, 17, 23). Dyspnea appears due to pulmonary edema and/or pleural effusion. Pulmonary edema develops from increased left ventricular filling pressures, secondary to left ventricular diastolic dysfunction and mitral regurgitation, which is the most frequent and pernicious complication of cardiomyopathies.

Pericardial effusion in cats is relatively rare, and the most common underlying cause of pericardial effusion is congestive heart failure (induced by cardiomyopathies, mitral valve disease, or pulmonic and aortic stenosis) and feline infectious peritonitis (37, 38), followed by neoplasia, pericardial–peritoneal diaphragmatic hernia, systemic infections, and uremia (37, 39, 40). Pleural effusion in cases of HCM indicates a biventricular failure (41). Right ventricular failure in the setting of AS may be secondary to pulmonary venous and arterial hypertension, caused by elevated left atrial pressures (16). Therefore the pleural effusion presented here most probably was related with the congestive heart failure caused by the myocardial hypertrophy rather than coronavirus (feline infectious peritonitis). One cat had pericardial effusion, diagnosed postmortem with SAS (16), with no evidence of feline coronavirus infection.

Another possible complication of SAS is thromboembolism. There is only one case that developed it (23), and it had specific clinical signs: hindlimbs paralysis with no palpable femoral pulses, cyanosis of the footpads, and excessive vocalization. Even though our cat presented a severely enlarged LA, on echocardiography and necropsy, there were no thrombus identified.

Stepien and Bonagura (16) consider a high risk of bacterial endocarditis in SAS and recommend antibiotic therapy, although this complication has not been reported so far in feline fixed SAS. On histopathological examination of the endocardium and the structure that produced the stenosis, there was no evidence of bacteria involvement.

To the author’s best knowledge, this is the first case describing clinical signs, ECG, echocardiography, and histopathological findings in a cat with cushion-like pseudovalve subaortic stenosis.

## Data Availability

The raw data supporting the conclusions of this article will be made available by the authors upon request. Requests to access the datasets should be directed to constantin.cerbu@usamvcluj.ro.

## References

[1] Petrič AD Perovič A Švara T Dovč P. Aortic stenosis in dogs and cats: past, present and future In: Magnusson P, editor. Aortic stenosis-current perspectives. London, UK: IntechOpen (2019). 1–20.

[2] Schrope DP. Prevalence of congenital heart disease in 76,301 mixed-breed dogs and 57,025 mixed-breed cats. J Vet Cardiol. (2015) 17:192–202. doi: 10.1016/j.jvc.2015.06.001, PMID: 26363941

[3] Bussadori C Amberger C Le Bobinnec G Lombard CW. Guidelines for the echocardiographic studies of suspected subaortic and pulmonic stenosis. J Vet Cardiol. (2000) 2:15–22. doi: 10.1016/S1760-2734(06)70007-8, PMID: 19081330

[4] Tidholm A. Retrospective study of congenital heart defects in 151 dogs. J Small Anim Pract. (1997) 38:94–8. doi: 10.1111/j.1748-5827.1997.tb03326.x, PMID: 9097239

[5] Oliveira P Domenech O Silva J Vannini S Bussadori R Bussadori C. Retrospective review of congenital heart disease in 976 dogs. J Vet Intern Med. (2011) 25:477–83. doi: 10.1111/j.1939-1676.2011.0711.x, PMID: 21418326

[6] Pyle RL Patterson DF Chacko S. The genetics and pathology of discrete subaortic stenosis in the Newfoundland dog. Am Heart J. (1976) 92:324–34. doi: 10.1016/S0002-8703(76)80113-5, PMID: 986114

[7] Crofton AE Kovacs SL Stern JA. Subvalvular aortic stenosis: learning from human and canine clinical research. Cardiol Res. (2023) 14:319–33. doi: 10.14740/cr1547, PMID: 37936623 PMC10627371

[8] Kittleson MD Kienle RD. Small animal cardiovascular medicine. St. Louis, MO: Mosby (1998). 603 p.

[9] Zuluaga SA Aldana SN Gutiérrez MC Bustamante ZS Muñoz CGP Zuluaga MN. Left ventricular outflow tract obstruction. Rev Colomb Radiol. (2017) 28:4609–15.

[10] Scansen BA Schneider M Bonagura JD. Sequential segmental classification of feline congenital heart disease. J Vet Cardiol. (2015) 17, 10–52. doi: 10.1016/j.jvc.2015.04.005 26776571

[11] Margiocco ML Zini E. Fixed subaortic stenosis in a cat. Vet Rec. (2005) 156:712–4. doi: 10.1136/vr.156.22.712, PMID: 15923555

[12] Thomas WP Gaber CE Jacobs GJ Kaplan PM Lombard CW Moise NS . Recommendations for standards in transthoracic two-dimensional echocardiography in the dog and cat. Echocardiography committee of the specialty of cardiology, American College of Veterinary Internal Medicine. J Vet Intern Med. (1993) 7:247–52. doi: 10.1111/j.1939-1676.1993.tb01015.x, PMID: 8246215

[13] Slezak P Steinhart L Prochazka J Endrys J Jurin I. The angiographic appearances of subvalvar aortic stenosis. Br J Radiol. (1965) 38:350–5. doi: 10.1259/0007-1285-38-449-350, PMID: 14280287

[14] Prophet EB Mills B Arrington JB Sobin LH. (1992). Laboratory methods in Histotechnology. Washington, DC: Armed Forces Institute of Pathology-American Registry of Pathology; 29–58, 132-133.

[15] Maxie MG. Jubb, Kennedy, and palmer's pathology of domestic animals, vol. 3. 6th ed. St. Louis, Missouri: Elsevier (2016).

[16] Stepien RL Bonagura JD. Aortic stenosis: clinical findings in six cats. J Small Anim Pract. (1991) 32:341–50. doi: 10.1111/j.1748-5827.1991.tb00945.x

[17] Watson CE Payne JR Borgeat K. Valvular aortic stenosis in three cats. J Vet Cardiol. (2019) 25:1–6. doi: 10.1016/j.jvc.2019.06.005, PMID: 31437784

[18] Liu SK. Pathology of feline heart diseases. Vet Clin North Am. (1977) 7:323–39. doi: 10.1016/s0091-0279(77)50033-0, PMID: 325872

[19] Severin GA. Congenital and acquired heart disease. J Am Vet Med Assoc. (1967) 151:1733–6.

[20] Bolton GR Liu SK. Congenital heart diseases of the cat. Vet Clin North Am. (1977) 7:341–53. doi: 10.1016/S0091-0279(77)50034-2, PMID: 867735

[21] Saperstein G Harris S Leipold HW. Congenital defects in domestic cats. Feline Pract. (1976) 6:18–43.

[22] Sousa MG Pascon JPE Santos PAC Camacho AA. WSAVA- Worls small animal veterinary assoc. editor severe aortic stenosis in a Persian kitten In: World small animal veterinary association world congress proceedings. São Paulo State University, Brazil: (2008)

[23] Ferreira AM Stedile ST d O Silva VBC Souza MG. Arterial thromboembolism secondary to subaortic stenosis in a Persian kitten. Acta Sci Vet. (2018) 46:5. doi: 10.22456/1679-9216.86826

[24] Tidholm A Ljungvall I Michal J Haggstrom J Hoglund K. Congenital heart defects in cats: a retrospective study of 162 cats (1996-2013). J Vet Cardiol. (2015) 17:S215–9. doi: 10.1016/j.jvc.2014.09.004, PMID: 26776580

[25] Szatmári V. Congenital supravalvular aortic stenosis in a kitten. J Vet Cardiol. (2022) 41:227–30. doi: 10.1016/j.jvc.2022.04.001, PMID: 35567886

[26] Deutsch V Shem-Tov A Yahini JH Neufeld HN. Subaortic stenosis (discrete form). Classification and angiocardiographic features. Radiology. (1971) 101:275–86. doi: 10.1148/101.2.275, PMID: 5114765

[27] Vogt J Dische R Rupprath G de Vivie ER Kotthoff S Kececioglu D. Fixed subaortic stenosis: an acquired secondary obstruction? A twenty-seven year experience with 168 patients. Thorac Cardiovasc Surg. (1989) 37:199–206. doi: 10.1055/s-2007-1020318, PMID: 2799791

[28] Ferasin L. Feline myocardial disease. 1: classification, pathophysiology and clinical presentation. J Feline Med Surg. (2009) 11:3–13. doi: 10.1016/j.jfms.2008.11.008, PMID: 19154970 PMC11135475

[29] Fujii Y Masuda Y Takashima K Ogasawara J Machida N Yamane Y . Hypertrophic cardiomyopathy in two kittens. J Vet Med Sci. (2001) 63:583–5. doi: 10.1292/jvms.63.583, PMID: 11411510

[30] Fuentes VL Johnson L Dennis S. BSAVA manual of canine and feline cardiorespiratory medicine. 2nd ed. Quedgeley, Gloucestershire, United Kingdom: BSAVA (2010).

[31] Parto P Tadjalli M Ghazi SR. Macroscopic and microscopic studies on moderator bands in the heart of ostrich (*Stuthio camelus*). Global Veterinária. (2010) 4:374–9.

[32] Wray JD Gajanayake I Smith SH. Congestive heart failure associated with a large transverse left ventricular moderator band in a cat. J Feline Med Surg. (2007) 9:56–60. doi: 10.1016/j.jfms.2006.03.006, PMID: 16861023 PMC10911575

[33] Fox PR. Textbook of canine and feline cardiology, principle and clinical practice. Philadelphia, USA: WB Saunders (1999).

[34] Ferasin L. Feline cardiomyopathy. In Pract. (2012) 34:204–13. doi: 10.1136/inp.e2271

[35] Payne JR Brodbelt DC Luis FV. Cardiomyopathy prevalence in 780 apparently healthy cats in rehoming centres (the CatScan study). J Vet Cardiol. (2015) 17:S244–57. doi: 10.1016/j.jvc.2015.03.008, PMID: 26776583

[36] Côté E MacDonald KA Meurs KM Sleeper MM. Feline Cardiology. Chichester, West Sussex, United Kingdom: Wiley-Blackwell (2011).

[37] Davidson BJ Paling AC Lahmers SL Nelson OL. Disease association and clinical assessment of feline pericardial effusion. J Am Anim Hosp Assoc. (2008) 44:5–9. doi: 10.5326/0440005, PMID: 18175793

[38] Rush JE Keene BW Fox PR. Pericardial disease in the cat: a retrospective evaluation of 66 cases. J Am Anim Hosp Assoc. (1990) 26:39–46.

[39] Boon JA. Veterinary echocardiography. 2nd ed. Chichester, West Sussex, United Kingdom: Wiley-Blackwell (2010).

[40] Hall DJ Shofer F Meier CK Sleeper MM. Pericardial effusion in cats: a retrospective study of clinical findings and outcome in 146 cats. J Vet Intern Med. (2007) 21:1002–7. doi: 10.1111/j.1939-1676.2007.tb03056.x, PMID: 17939556

[41] Fox PR. Canine and feline cardiology. New York: Churchill Livingstone (1988). 676 p.

